# Comparing heatwave experiences, behaviors, and risk perceptions across high-risk populations in the Netherlands: A cross-sectional survey study

**DOI:** 10.1016/j.joclim.2026.100689

**Published:** 2026-05-16

**Authors:** Eline van de Kamp, Francine Schneider, Hein Daanen, Maud Huynen

**Affiliations:** a Department of Human Movement Sciences, Vrije Universiteit Amsterdam, De Boelelaan 1105, 1081 HV Amsterdam, the Netherlands; b Department of Health Promotion, Care and Public Health Research Institute CAPHRI, Maastricht University, 6200 MD Maastricht, the Netherlands; c Maastricht Sustainability Institute (MSI), Maastricht University, 6200 MD Maastricht, the Netherlands

**Keywords:** Heat waves, Risk perception, Heat adaptive behaviors, Vulnerable populations, Health risk, Climate change

## Abstract

**Introduction:**

Heatwaves are a growing public health concern in the Netherlands, particularly for high-risk populations. To reduce heat-related health risks and increase the adoption of protective behaviors, more insight is needed into how different populations experience and manage heat. This study explores how high-risk groups in the Netherlands experienced a heatwave, how they perceived heat-related risks, and the extent of protective measures they took.

**Methods:**

A cross-sectional online survey was conducted among older adults (50+) directly following a heatwave in July 2023 in Limburg, the Netherlands. High-risk groups were defined from the sample (n = 514) based on age (≥65 years), gender (female), health status (poor/fair self-rated health, chronic illness, or medication use), socioeconomic position (low or lower-middle income), and social isolation (self-rated social contact score ≤3/10). Quantitative analyses assessed associations between risk characteristics, heat experiences, protective behaviors, risk perception, and response efficacy.

**Results:**

Heat was not experienced or managed uniformly. Lower health status and socioeconomic position were most strongly associated with discomfort, emotional strain, and health issues. Individuals with lower socioeconomic position adopted more protective behaviors but mostly relied on low-cost measures. Those with lower health status showed no increase in protective behaviors, possibly due to physical constraints. Adverse heat-related outcomes increased with the number of high-risk factors, indicating cumulative vulnerability. Despite this, risk perception remained low across all groups.

**Conclusion:**

Individuals' experiences and perceptions were most strongly associated with socioeconomic and health-related disadvantages. Given the compounding nature of these vulnerabilities and low risk perception, targeted interventions tailored to lived realities are essential.

## 1 Introduction

Heatwaves are becoming a growing public health concern as their frequency, intensity, and duration are rising due to climate change [1,2]. These extreme heat events are associated with excess mortality [[3], [4], [5]], particularly cardiovascular, respiratory, renal, and other chronic disease mortality [[6], [7], [8]], as well as increased hospital admissions [9,10] and mental health issues [[11], [12], [13]]. Although heatwaves affect entire populations, their health impacts are not equally distributed. The elderly are the most widely recognized at-risk group, due to declines in thermoregulation [14,15], reduced thirst sensation [16], and a higher prevalence of chronic conditions and medication use [[17], [18], [19], [20]]. Older women may encounter greater health risks than men, although underlying causes remain unclear [[21], [22], [23]]. Socio-environmental factors can further increase heat-related health risks. Individuals experiencing social isolation may lack access to information and support networks [24,25], while individuals with lower socioeconomic position (SEP) may have limited access to cooling resources, including air-conditioning, and live in poorer housing conditions [22,24,26]. These vulnerabilities often co-exist, jointly increasing heat-related exposure and sensitivity while reducing adaptive capacity [24,27].

Traditionally, heat vulnerability has been assessed using these risk factors related to exposure, sensitivity, and adaptive capacity [28]. While these approaches are essential for identifying high-risk populations, they provide limited insight into how heat is actually experienced, perceived, and managed. More recently, increasing attention is being given to the subjective dimensions of heat risk, including perceived (dis)comfort, self-reported health symptoms, perceived personal health risk, and beliefs about the effectiveness of protective measures [[29], [30], [31]]. These subjective dimensions are particularly relevant as they might play a role in whether an individual adopts protective behaviors, which in turn can mitigate or prevent heat-related morbidity and mortality [32]. While an increasing number of heat-health warning systems, emergency preparedness plans, and behavioral advice campaigns are being launched [33,34], the effectiveness of these efforts in promoting protective actions among diverse high-risk populations remains limited [[35], [36], [37]]. Most of these interventions focus on the general population or certain high-risk groups, but do not account for overlapping vulnerabilities and lived realities [37,38].

This is also the case for the Netherlands, a country historically unaccustomed to heatwaves but increasingly affected with record-breaking temperatures exceeding 40 °C in recent years [39]. Although Dutch epidemiological studies have shown increases in all-cause mortality and healthcare utilization during heatwaves, especially among older adults [[40], [41], [42], [43]], and the Dutch National Heatwave Plan explicitly identifies and targets high-risk groups [44], little is known about how individuals in these groups experience heat, perceive risks, and adopt protective behaviors.

The current exploratory study seeks to address these gaps by examining heat-related discomfort, emotional experiences, health symptoms, risk perception, perceived response efficacy, and adoption of protective behaviors during a regional 2023 heatwave in the province of Limburg, the Netherlands. Over the past decades, Limburg has experienced the highest number of tropical days (≥30 °C) among all Dutch provinces [45], making it particularly relevant for studying heat-related health impacts. Moreover, this region includes a substantial representation of populations considered at higher risk during heat events, as demographically, Limburg exceeds the national average in both the proportion of individuals older than 65 years [46] and households with lower incomes [47]. Together, these characteristics make the region an optimal setting for studying heatwaves among a relatively high representation of various high-risk groups.

This study focuses on five different high-risk groups: individuals of older age, of female gender, with lower health status, with lower SEP, and with limited social contact. Specifically, this study aims to address three research questions:

• Do individuals in the defined high-risk groups differ from low-risk individuals in heat-related experiences and protective behaviors?
• Is belonging to multiple high-risk groups associated with greater differences in these experiences and behaviors?
• How are risk perception (i.e., the belief that one is personally at risk of heat-related health problems) and response efficacy (i.e., the belief that recommended protective actions can effectively reduce the health threat [48]) associated with heat-related experiences and protective behaviors? With the insights from this exploratory study, we aim to provide a foundation for more targeted and tailored public health responses to reduce heat-related health risks across populations in the Netherlands.

## 2 Methods

### 2.1 Study design and sample

A cross-sectional survey was conducted directly following a regional heatwave in the Dutch province of Limburg. The survey was part of a broader project on heat and its effects on health and wellbeing [49]. This study focused on a subset of survey questions related to experiences, behaviors, and perceptions among high- and low-risk groups during the heatwave.

The study sample (n = 514) included residents of Limburg aged 50 and older, recruited through an ISO-certified internet panel (ISO-26362) managed by Flycatcher Internet Research [50]. The study complied with the ethical guidelines of Vrije Universiteit Amsterdam and Maastricht University and adhered to The Code of Ethics of the World Medical Association (Declaration of Helsinki). Informed consent was obtained from all participants.

### 2.2 Data collection

The heatwave was regional, occurring in the province of Limburg from July 7 to 11, 2023. It met the official Dutch definition of a heatwave (five consecutive days >25 °C, including three days >30 °C), indicating a prolonged period of high temperatures. Data were collected from July 12 to 19 using an online questionnaire. The survey assessed the presence of five risk factors (age, gender, health status, SEP, and social isolation) to classify participants into high- or low-risk groups (Table 1).

**Table 1:** Criteria for high-risk group classifications. Definitions used to identify participants as belonging to one or more heat-vulnerable high-risk groups. *See Supplementary Material 1 for included chronic conditions and medications.

Risk Factor	Criteria for High-Risk Group
Age	65 years and older
Gender	Female
Health Status	At least one of the following:
	• Self-reported health status of *fair* or *poor*
	• Presence of one or more chronic illnesses*
	• Use of medication*
Socioeconomic Position (SEP)	Annual gross household income falling within the low or lower-middle income brackets based on Statistics Netherlands data [53]
Social isolation	Score of 3 or less on a 0 to 10 scale assessing frequency of social contact with family, friends, neighbors, and acquaintances

To explore the association between compounded vulnerability and heat-related outcomes, a multiple-risk index was calculated by summing the number of high-risk characteristics of each participant (ranging from 0 to 5). This unweighted additive index represents a simplification rather than a validated measure of heat vulnerability, as different risk factors contribute to vulnerability in distinct ways. It follows prior cumulative risk frameworks in public health (e.g., [51,52]).

Additionally, the survey assessed the following key measures:

• **Heat-related discomfort, including emotional experience:** included one item on the general level of discomfort during the heatwave (0–10 scale) and five items on specific emotional experiences during the heatwave (feeling less/more happy, calm, nervous, irritated, and gloomy) (5-point Likert scales).
• **Heat-related health issues:** included level of health concern (0–10 scale), changes in occurrence of health issues, such as fatigue or respiratory problems (5-point Likert scales), and heat-related medical help-seeking (yes/no). For participants reporting chronic conditions (n = 180), symptom worsening was also assessed (0–10 scale). The specific health issues in this category were based on the heat-related health issues mentioned in the Dutch National Heatwave Plan [54].
• **Protective measures taken during the heatwave:** included self-rated extent of protective measures taken (0–10 scale) and frequencies of specific behaviors, such as using air-conditioning or staying indoors (5-point Likert scales). The included heat-protective behaviors were identified from the general public health recommendations by the Dutch National Heatwave Plan, the Dutch Red Cross, the Municipal Public Health Services (GGD), and the National Institute for Public Health and the Environment (RIVM) [[54], [55], [56], [57]].
• **Risk perception and response efficacy:** included agreement with statements on risk perception (“I am at greater risk of health problems from heat than other people”) and response efficacy (“There is little that can be done to prevent or reduce heat-related health issues during a heatwave”) (5-point Likert scales).

A detailed list of included survey questions and answer options is provided in Supplementary Material 1, and details on variable coding and analyses in Supplementary Material 2.

### 2.3 Statistical analyses

Descriptive statistics summarized participant demographics and survey responses. Three main research aims guided the analysis. Firstly, unadjusted differences in discomfort, health issues, and protective behaviors were compared between high-risk and low-risk groups using independent *t*-tests, Pearson’s chi-square tests, and Mann-Whitney U tests. Multivariate regression then assessed the effect of single risk factors, while controlling for the others. Secondly, univariate regression tested associations between the multiple-risk index (number of high risk groups an individual was assigned to; range 0–5) and discomfort, health issues, and protective behaviors. Thirdly, multivariate regression explored how risk perception and response efficacy relate to the heat-related outcomes, adjusting for the five risk factors. Statistical significance was set at *p* *<* 0.05. Analyses were conducted using R (version 4.4.3). The results section mainly highlights significant outcomes from the regression models.

## 3 Results

### 3.1 Descriptive results

Table 2 summarizes participant characteristics. Of the 514 participants, 53.1 % were individuals of older age (≥65), 51.6 % were female gender, 75.9 % reported lower health status, 36.6 % reported lower socioeconomic position (SEP), and 5.6 % experienced social isolation. Nearly all participants fell into at least one high-risk group: 19.6 % were assigned to one group, 33.5 % to two, 31.1 % to three, and 10.7 % to four, with none meeting all five high-risk criteria.

**Table 2:** Demographic characteristics and high-risk classification of survey participants (n = 514). Asterisks (*) indicate the high-risk group.

Variable	n (%)
**Age**	
50–64 years	241 (46.9 %)
≥65 years*	273 (53.1 %)
**Gender**	
Male	249 (48.4 %)
Female*	265 (51.6 %)
**Health Status**	
No health risk factors	124 (24.1 %)
Fair/poor health, chronic illness, or medication use*	390 (75.9 %)
**Socioeconomic Position**	
Low or lower-middle income*	188 (36.6 %)
Middle, upper-middle or high income	326 (63.4 %)
**Social Isolation**	
Self-reported social contact score of ≤3/10*	29 (5.6 %)
Self-reported social contact score of >3/10	485 (94.4 %)
**Multiple-Risk Index (number of high-risk groups)**	
0	26 (5.1 %)
1	101 (19.6 %)
2	172 (33.5 %)
3	160 (31.1 %)
4	55 (10.7 %)
5	0 (0.0 %)

### 3.2 Risk factors associated with heat-related outcomes

#### 3.2.1 Heat-related discomfort

**Fig. 1:**
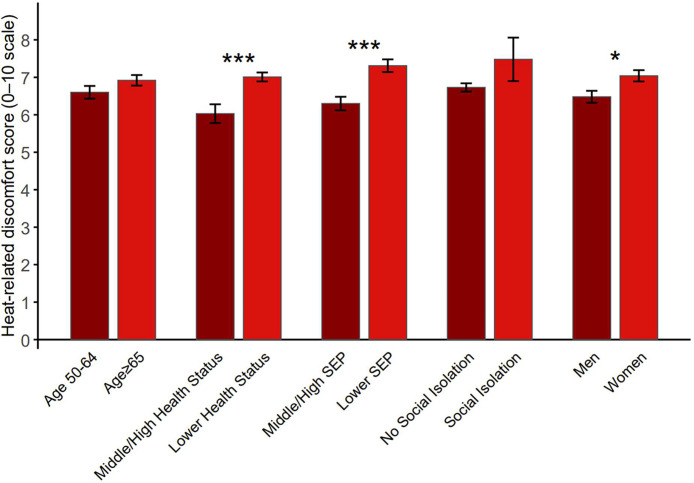
Reported discomfort on a 0–10 scale by risk factor classification (independent samples *t*-test). Within each risk factor, bars on the right show the high-risk group. Mean± SE are shown. p < 0.05: *; p < 0.01: **; p < 0.001: ***. SEP: socioeconomic position.

Overall heat-related discomfort was higher in the high-risk groups compared to the low-risk groups across all five risk factors, with significant differences among individuals with lower health status (7.01 vs. 6.03, *p* *<* 0.001), lower SEP (7.31 vs. 6.30, *p* *<* 0.001), and women (7.04 vs. 6.48, *p* *=* 0.011) (Fig. 1). When controlling for all risk factors, lower health status (b = 0.98; CI: 0.40–1.56; *p* *=* 0.001) and SEP (b = 0.81; CI: 0.30–1.33; *p* *=* 0.002) remained significantly associated with higher discomfort.

**Fig. 2:**
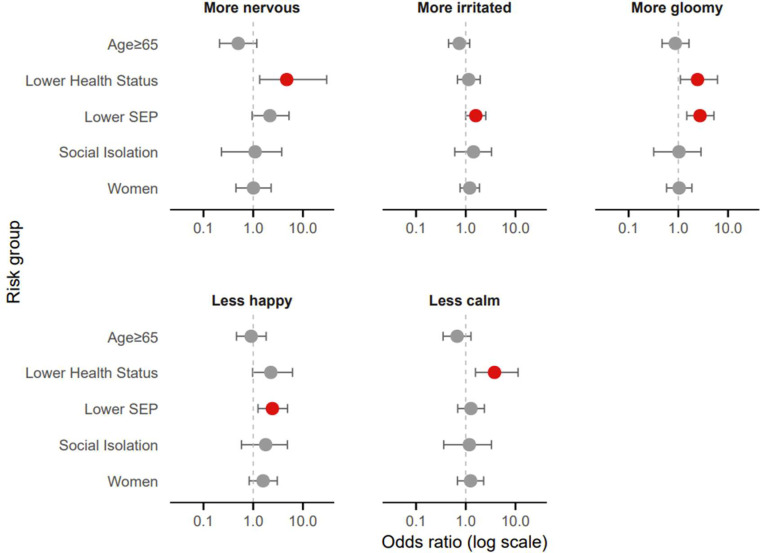
Adjusted odds ratios for emotional experience by risk factor (multivariate logistic regression including all five risk groups). Red dots indicate significant odds ratios. SEP: socioeconomic position.

Emotional experiences during the heatwave varied across risk groups (Fig. 2). Multivariate regression analyses showed that individuals with lower health status felt significantly more nervous (OR=4.72, 95 % CI: 1.35–30.01, *p* *=* 0.039), gloomy (OR=2.43, 95 % CI: 1.10–6.13, *p* *=* 0.040), and less calm (OR=3.78, 95 % CI: 1.57–11.28, *p* *=* 0.007). Individuals with lower SEP were significantly more likely to feel irritated (OR=1.59, 95 % CI: 1.00–2.53, *p* *=* 0.049), gloomy (OR=2.73, 95 % CI: 1.48–5.21, *p* *=* 0.002), and less happy (OR=2.43, 95 % CI: 1.25–4.91, *p* *=* 0.010).

#### 3.2.2 Heat-related health issues

**Fig. 3:**
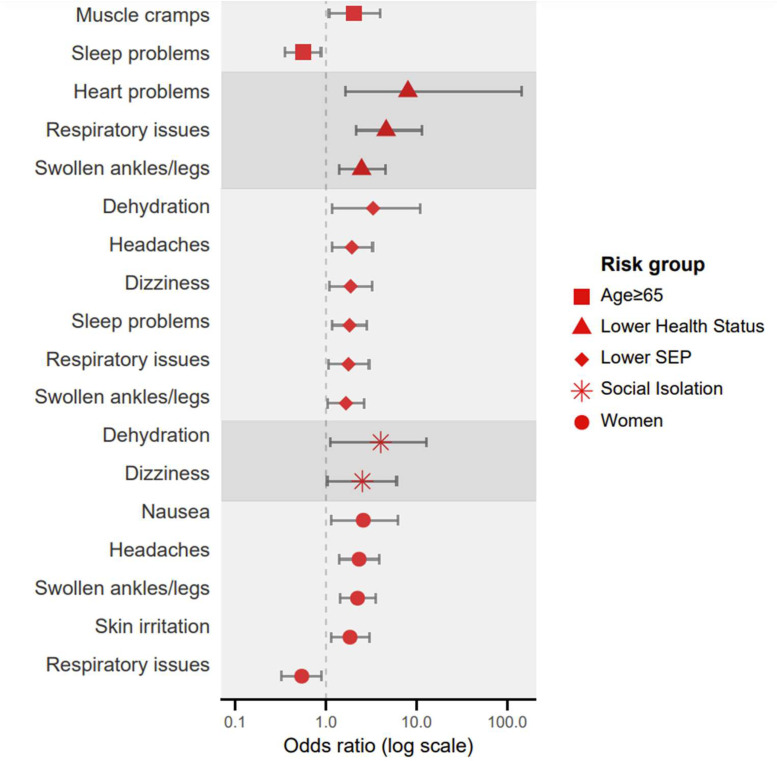
Adjusted odds ratio for heat-related health issues by risk factor (multivariate logistic regression including all five risk groups). Only significant odds ratios are shown. SEP: socioeconomic position.

Participants reported an increase in various health issues during the heatwave (Fig. 3). Lower health status was significantly associated with swollen ankles (OR=2.47, 95 % CI: 1.39–4.42, *p* *=* 0.002), heart problems (OR=8.02, 95 % CI: 1.06–153.41, *p* *=* 0.044), and respiratory issues (OR=4.61, 95 % CI: 2.37–9.73, *p* *<* 0.001). Moreover, it was linked to greater health concern during the heatwave (b = 1.24, 95 % CI: 0.63–1.85, *p* *<* 0.001).

Lower SEP was significantly associated with dizziness (OR=1.87, 95 % CI: 1.10–3.23, *p* *=* 0.021), respiratory problems (OR=1.77, 95 % CI: 1.06–2.99, *p* *=* 0.028), dehydration (OR=3.31, 95 % CI: 1.11–11.87, *p* *=* 0.032), swollen ankles (OR=1.66, 95 % CI: 1.05–2.65, *p* *=* 0.030), headaches (OR=1.93, 95 % CI: 1.15–3.32, *p* *=* 0.013), and sleep problems (OR=1.82, 95 % CI: 1.17–2.88, *p* *=* 0.008). In addition, it was linked to greater health concern (b = 1.45, 95 % CI: 0.91–1.99, *p* *<* 0.001) and worsening of chronic disease symptoms (b = 1.57, 95 % CI: 0.80–2.34, *p* *<* 0.001). Seeking medical help was also more likely among individuals in lower SEP (OR=2.88, CI: 1.10–8.47, *p* *=* 0.039).

Social isolation increased the odds of dizziness (OR=2.52, 95 % CI: 1.05–6.39, *p* *=* 0.039) and dehydration (OR=4.02, 95 % CI: 1.21–15.18, *p* *=* 0.023). Women more frequently reported swollen ankles (OR=2.23, 95 % CI: 1.55–3.21, *p* *<* 0.001), headaches (OR=2.32, 95 % CI: 1.42–3.85, *p* *=* 0.001), skin irritation (OR=1.84, 95 % CI: 1.13–3.01, *p* *=* 0.014), and nausea (OR=2.58, 95 % CI: 1.13–6.11, *p* *=* 0.026), but less frequently reported respiratory problems (OR=0.54, 95 % CI: 0.32–0.89, *p* *=* 0.018). Age showed limited associations, though older age was linked to fewer sleep problems (OR=0.56, 95 % CI: 0.36–0.86, *p* *=* 0.013) and more muscle cramps (OR=2.04, 95 % CI: 1.06–4.09, *p* *=* 0.032).

#### 3.2.3 Protective measures taken

**Fig. 4:**
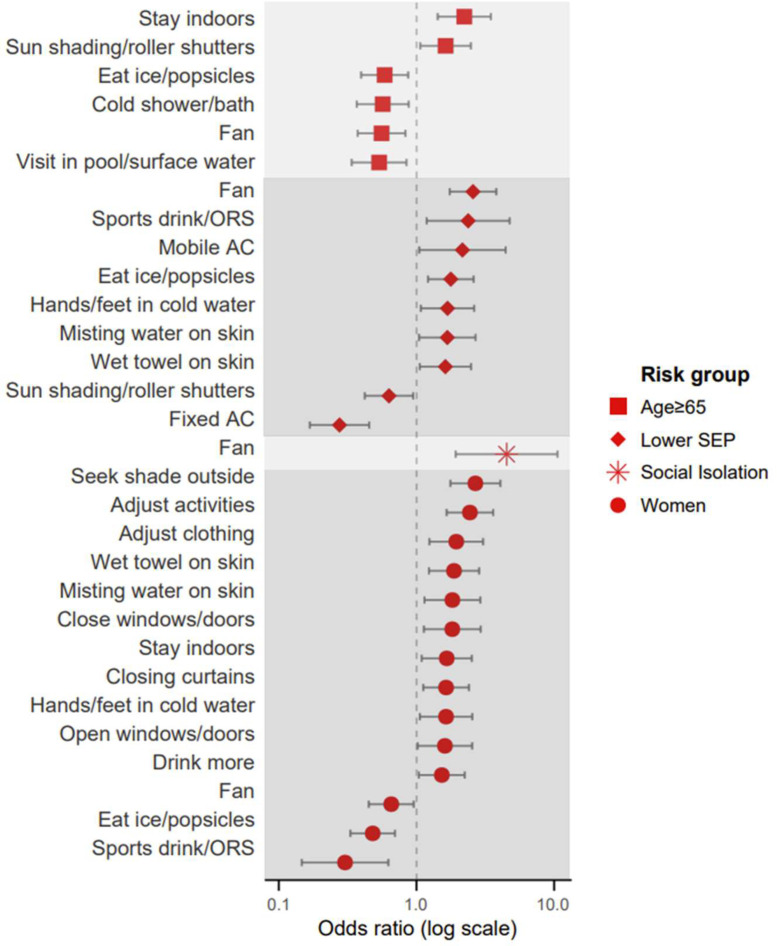
Adjusted odds ratio for frequency of protective measures taken during the heatwave by risk factor (multivariate ordinal logistic regression including all five risk groups). Only significant odds ratios are shown. SEP: socioeconomic position. The risk factor ‘lower health status’ did not show any significant associations and is not included in the figure.

Compared to individuals aged 50–64, those aged ≥65 were more likely to stay indoors (OR=2.22; 95 % CI: 1.43–3.47; *p* *<* 0.001) and use external sun shading (OR=1.63; 95 % CI: 1.07–2.48; *p* *=* 0.024), but less likely to adopt more active protective behaviors, such as taking cold showers (OR=0.57; 95 % CI: 0.37–0.88; *p* *=* 0.011) or seeking outdoor surface water (OR=0.54; 95 % CI: 0.34–0.85; *p* *=* 0.008) (Fig. 4). Additionally, women more frequently reported seeking shade (OR=2.68; 95 % CI: 1.76–4.07; *p* *<* 0.001), adjusting daily activities (OR=2.45; 95 % CI: 1.66–3.61; *p* *<* 0.001), and staying indoors (OR=1.66; 95 % CI: 1.09–2.51; *p* *=* 0.018), and adopted low-cost strategies such as using wet towels (OR=1.87; 95 % CI: 1.23–2.85; *p* *=* 0.003), misting water (OR=1.82; 95 % CI: 1.14–2.91; *p* *=* 0.012), and cold footbaths (OR=1.64; 95 % CI: 1.06–2.54; *p* *=* 0.027). Similarly, individuals with lower SEP more often used these low-cost strategies, including wet towels (OR=1.62; 95 % CI: 1.06–2.49; *p* *=* 0.027), cold footbaths (OR=1.68; 95 % CI: 1.08–2.63; *p* *=* 0.022), misting water (OR=1.68; 95 % CI: 1.04–2.69; *p* *=* 0.032), and fans (OR=2.57; 95 % CI: 1.74–3.79; *p* *<* 0.001), but were less likely to use fixed air-conditioning (OR=0.28; 95 % CI: 0.17–0.45; *p* *<* 0.001) or external sun shading (OR=0.63; 95 % CI: 0.42–0.95; *p* *=* 0.027). Socially isolated individuals were significantly more likely to use a fan (OR=4.52; 95 % CI: 1.93–10.59; *p* *<* 0.001). No significant associations were found between health status and specific protective behaviors.

### 3.3 Multiple-risk index

**Fig. 5:**
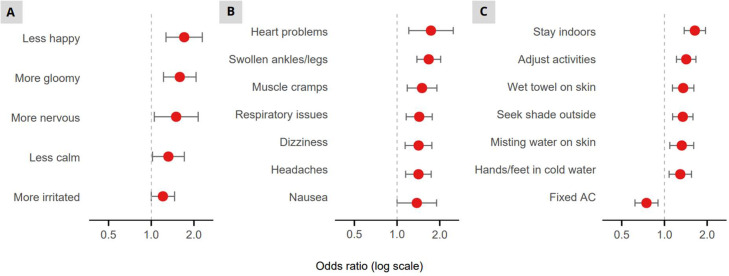
Crude odds ratios of heat-related outcomes by multiple-risk index (univariate logistic regression). (A) Emotional experiences; (B) Health issues; (C) Frequency of protective measures taken. Only significant odds ratios are shown.

Participants with a higher multiple-risk index reported greater discomfort (b = 0.59; CI: 0.38–0.79; *p* *<* 0.001), greater health concern (b = 0.77; CI: 0.55–0.98; *p* *<* 0.001), and worsening of chronic conditions (b = 0.75; CI: 0.38–1.12; *p* *<* 0.001) during the heatwave. A higher multiple-risk index was significantly associated with more irritation (OR=1.21, 95 % CI: 1.00–1.46, *p* *=* 0.047), more gloominess (OR=1.59, 95 % CI: 1.22–2.07, *p* *<* 0.001), more nervousness (OR=1.50, 95 % CI: 1.05–2.14, *p* *=* 0.025), less happiness (OR=1.71, 95 % CI: 1.27–2.29, *p* *<* 0.001) and less calmness (OR=1.32, 95 % CI: 1.02–1.71, *p* *=* 0.036) (Fig. 5a). Similarly, participants with a higher multiple-risk index showed an increase in heat-related health issues, among which swollen ankles (OR=1.67, 95 % CI: 1.38–2.03, *p* *<* 0.001), dizziness (OR=1.42, 95 % CI: 1.14–1.76, *p* *=* 0.002), heart problems (OR=1.73, 95 % CI: 1.21–2.49, *p* *=* 0.003), headaches (OR=1.42, 95 % CI: 1.15–1.74, *p* *<* 0.001), and respiratory issues (OR=1.43, 95 % CI: 1.16–1.77, *p* *<* 0.001) (Fig. 5b).

Regarding protective behaviors, those with a higher multiple-risk index were less likely to use fixed air-conditioning (OR=0.75; 95 % CI: 0.62–0.90; *p* *=* 0.002) but more likely to adopt low-cost strategies, including misting water (OR=1.33; 95 % CI: 1.09–1.61; *p* *=* 0.004), wet towels (OR=1.36; 95 % CI: 1.14–1.61; *p* *<* 0.001), staying indoors (OR=1.64; 95 % CI: 1.38–1.95; *p* *<* 0.001), seeking shade (OR=1.35; 95 % CI: 1.14–1.59; *p* *<* 0.001), and adjusting daily activities (OR=1.42; 95 % CI: 1.22–1.67; *p* *<* 0.001) (Fig. 5c).

### 3.4 Risk perception and perceived response efficacy

**Fig. 6:**
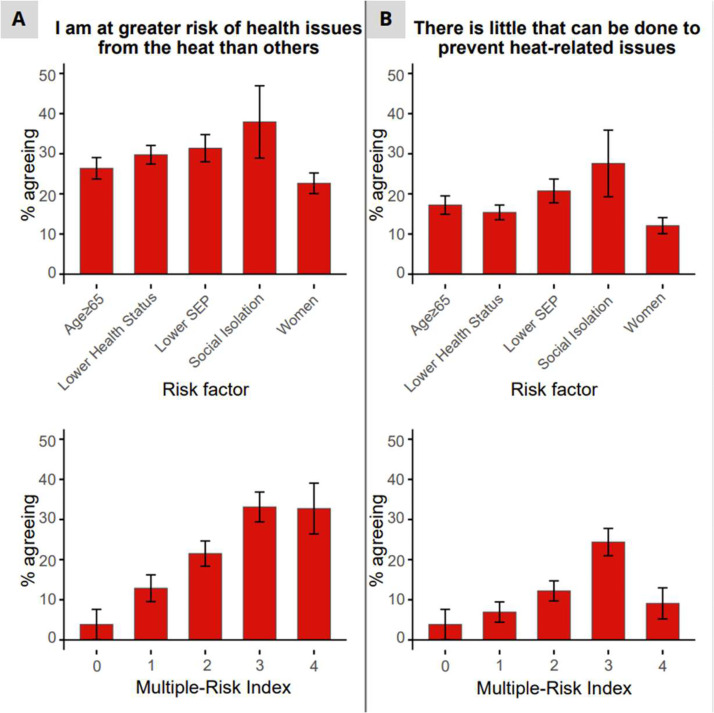
Percentage of respondents agreeing with statements about (A) risk perception and (B) response efficacy, by individual risk groups and multiple risk-index. Mean± SE are shown. SEP: socioeconomic position. Multiple-risk index: the number of high risk groups an individual was assigned to.

**Fig. 7:**
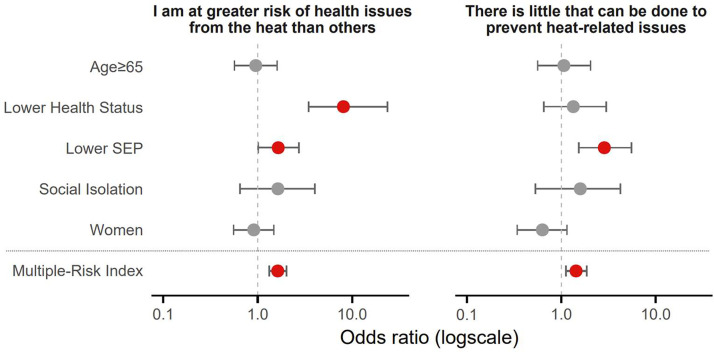
Adjusted odds ratio for risk perception and response efficacy by risk group (multivariate logistic regression including all five risk groups) and by multiple-risk index (univariate logistic regression). Red dots indicate significant odds ratios. SEP: socioeconomic position. Multiple-risk index: the number of high risk groups an individual was assigned to.

Across all high-risk groups, relatively few participants perceived themselves as more vulnerable to heat-related health issues than others: only 23 % of women, 26 % of individuals of older age, 30 % of individuals with lower health status, 31 % with lower SEP, and 38 % of individuals with limited social contact. Even among participants with three to four high-risk characteristics, only one-third considered themselves at increased risk (Fig. 6a). Multivariate regression showed that lower health status (OR=8.09; 95 % CI: 3.47–23.69; *p* *<* 0.001), lower SEP (OR=1.65; 95 % CI: 1.00–2.72; *p* *=* 0.048), and a higher multiple-risk index (OR=1.63; 95 % CI: 1.32–2.02; *p* *<* 0.001) were significantly associated with higher risk perception (Fig. 7).

Regarding response efficacy, the belief that little could be done to prevent or reduce heat-related health issues was also relatively uncommon across high-risk groups: 12 % of women, 15 % of individuals with lower health status, 17 % of individuals of older age, 21 % of individuals with lower SEP, and 28 % of individuals with limited social contact (Fig. 6b). In multivariate regression, lower SEP (OR=2.85; 95 % CI: 1.52–5.53; *p* *=* 0.001), and a higher multiple-risk index (OR=1.43; 95 % CI: 1.11–1.85; *p* *=* 0.002) were significantly associated with lower response efficacy (Fig. 7).

**Fig. 8:**
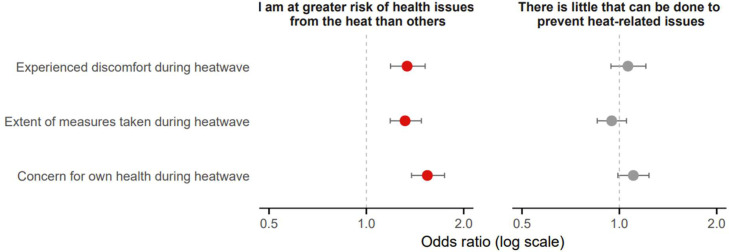
Adjusted odds ratios for risk perception and response efficacy by heat-related outcomes (multivariate logistic regression including all five risk groups). Red dots indicate significant odds ratios.

Perceiving oneself at greater risk was significantly associated with greater discomfort (OR=1.34; 95 % CI: 1.19–1.52; *p* *<* 0.001), greater health concern (OR=1.54; 95 % CI: 1.38–1.75; *p* *<* 0.001), and a higher self-reported extent of protective measures taken (OR=1.32; 95 % CI: 1.19–1.48; *p* *<* 0.001), while the perception that little can be done to reduce heat-related health issues was not significantly linked to these outcomes (Fig. 8).

## 4 Discussion

### 4.1 Main findings

This study explored how different high-risk groups in Limburg, the Netherlands, experienced the 2023 heatwave, the extent to which they adopted protective behaviors, and their perceptions of heat-related risks and response efficacy. Our findings confirm that heat is not experienced or managed equally across populations, underscoring the importance of incorporating subjective experiences alongside objective risk classifications when developing heat adaptation strategies.

Individuals with multiple risk factors experienced more adverse outcomes during the heatwave, including more discomfort, negative emotional experiences, and health issues. While heatwave interventions often target the general population or vulnerable groups primarily defined by age [37], our results – controlling for age – demonstrate that lower health status and SEP were most strongly associated with negative subjective experiences.

Individuals with lower health status reported more discomfort, negative emotional experiences, and heart and respiratory problems. Yet, they did not report more protective behaviors. This aligns with Bourret Soto and Guillon [58], who found no link between health status and heat-adaptive behaviors. Health-related constraints, such as reduced mobility, may hinder the ability to take protective actions or seek cooler environments [37], highlighting the need for interventions that accommodate these constraints.

In contrast, individuals with lower SEP, while also experiencing more discomfort and health issues, reported more protective behaviors. These mainly included low-cost measures, whereas air-conditioning and external shading were used less often, which may partly reflect the reliance on more affordable strategies. This group also more often believed that “little could be done” to reduce heat-related health issues. Reasons for this may be limited control over housing conditions and less access and use of air-conditioning [[59], [60], [61]]. These barriers can contribute to a sense of helplessness and lower perceived response efficacy.

Overall, reported risk perception was low. Even among the group with the most high-risk factors, only one-third considered themselves at greater risk than other people for heat-related health issues. This optimistic bias, the tendency to downplay one’s own vulnerability relative to others [62], contrasts with findings from hotter regions like Guangdong, China, and Phoenix, USA, where 85–90 % of residents perceived heatwaves as serious threats to their health [63,64]. Dutch residents may underestimate heat risks due to the country’s milder climate. Yet, the minimum mortality temperature in the Netherlands, the temperature at which mortality risk is lowest, is around 17.4 °C, far lower than in regions such as East Asia (∼25 °C) [65]. This means that what seems like moderate heat elsewhere can still pose serious health risks in the Netherlands. This study also found that risk perception was associated with the adoption of more protective behaviors. This aligns with findings from a recent meta-analysis in which a positive association was found between perceived threat and heat adaptive behavior [58]. However, neither the current study nor any of the included studies in the meta-analysis included longitudinal data, preventing any conclusions about causality. The observed association may be bidirectional or influenced by factors not measured in the current study, and future studies using a longitudinal designs are needed. Risk perception was also linked to greater discomfort and concern. While again no statements about causality can be made for this association, combining risk communication with practical tailored strategies to reduce heat risks may help avoid possible unintended consequences of raising anxiety and concern levels.

### 4.2 Strengths and limitations

A major strength of this study is the timing of data collection, which occurred immediately after the heatwave, minimizing recall bias [66]. However, several limitations remain. First, although the sample was representative of the 50+ population of Limburg by gender, age, education level, and region (Supplementary Material 3), the restriction to adults aged 50+ limits the generalizability of the findings to the younger Dutch population, who may differ in heat experiences, risk perception, and adoption of protective measures. In addition, the use of an online panel could have excluded individuals with low digital literacy [67], a group that may manage heat differently. Alternative strategies, such as paper-based surveys, could improve their inclusion. Second, all key variables in this study are self-reported, making measures of protective behavior prone to social desirability bias and measures of poor health or lower SEP susceptible to underreporting, which could lead to misclassifying high-risk individuals as low-risk. Third, while individuals experiencing social isolation reported strong adverse outcomes, their small number (5.6 %) limited statistical power to detect significant associations. Oversampling this group in future research could help better capture their experiences. Finally, the findings are context-specific to a single Dutch province and reflect climate, social, and housing conditions characteristic of the Netherlands. Therefore, caution is warranted when extrapolating results to different settings. The limitations suggest that some of the most vulnerable individuals may be underrepresented and that this exploratory study may underestimate disparities in heatwave impacts and responses. Nonetheless, its findings offer a valuable foundation for public health strategies and future research.

### 4.3 Implications and recommendations

This study highlights the need for tailored strategies targeting high-risk groups while taking subjective experiences of heat into account. Interventions should extend their focus to individuals with lower health status and SEP, providing financial support for effective but costlier cooling measures (e.g., external sun shading) and measures that accommodate possible health-related constraints. We recommend that actions aimed at increasing risk perception among high-risk individuals should also offer practical solutions to mitigate heat impacts. Importantly, interventions must account for overlapping vulnerabilities within high-risk groups as these can compound heat-related risks and adverse experiences. To ensure relevance and effectiveness, the perspectives of vulnerable groups should be central, ideally through co-creation and participatory approaches.

## 5 Conclusion

This study highlights the diverse subjective experiences and cumulative vulnerabilities of high-risk groups in Limburg, the Netherlands, during a heatwave. Socioeconomic and health-related disadvantages were most strongly associated with negative experiences and perceptions. Adverse outcomes increased with the number of high-risk factors, reflecting cumulative vulnerability. Combined with the low risk perception, even among those most at risk, these findings highlight the importance of integrating subjective experiences into heat adaptation strategies, informing targeted and tailored policies based on risk profiles.

## Declaration of generative AI and AI-assisted technologies in the writing process

During the preparation of this work the authors used ChatGPT (GPT-4) to improve readability. After using this tool, the authors reviewed and edited the content as needed and take full responsibility for the content of the published article.

## CRediT authorship contribution statement

**Eline van de Kamp:** Writing – review & editing, Writing – original draft, Visualization, Methodology, Formal analysis, Data curation. **Francine Schneider:** Writing – review & editing, Supervision, Methodology. **Hein Daanen:** Writing – review & editing, Methodology, Funding acquisition, Conceptualization. **Maud Huynen:** Writing – review & editing, Supervision, Methodology, Funding acquisition, Conceptualization.

## Declaration of competing interest

The authors declare that they have no known competing financial interests or personal relationships that could have appeared to influence the work reported in this paper.

## Appendix Supplementary materials

Image, application 1

Image, application 2

Image, application 3

## Footnotes

Supplementary material associated with this article can be found, in the online version, at doi:10.1016/j.joclim.2026.100689.
