# Supplementary material for: Comparing heatwave experiences, behaviors, and risk perceptions across high-risk populations in the Netherlands: A cross-sectional survey study
Source: J Clim Chang Health. 2026 May 16;29:100689. doi: 10.1016/j.joclim.2026.100689 (PMC13199810; doi:10.1016/j.joclim.2026.100689)
Supplement: Supplementary file 1 [file mmc1.docx]

**Supplementary Material 1 – Survey Components Included in the Analyses**

***1. Background variables and risk group criteria***

What is your age? [Open field: in years]

What is your gender?

- Male
- Female
- Other
- Prefer not to say

How would you describe your general health?

- Excellent
- Very good
- Good
- Fair
- Poor
- Prefer not to say

Do you have one or more chronic illnesses or conditions (e.g., high blood pressure, heart problems, diabetes, respiratory issues)?

- Yes, namely:
  - High blood pressure
  - Heart condition
  - Kidney disease
  - Diabetes
  - Respiratory issues (e.g., asthma, COPD, bronchitis)
  - Overweight/obesity
  - Mental health condition
  - Other, namely: [Open field]
- No, I do not have any chronic illnesses or conditions
- Prefer not to say

Which of the following types of medication do you use?

- Diuretics
- Medication for high blood pressure, other than diuretics
- Medication for heart conditions, other than diuretics
- Medication for kidney disease, other than diuretics
- Medication for diabetes
- Medication for respiratory issues (e.g., asthma, COPD, bronchitis)
- Medication for Parkinson’s disease
- Medication for psychosis
- Other, namely: [Open field]
- I do not use any medication
- Prefer not to say

What is your household’s gross annual income? (This includes all household members, including holiday pay and bonuses.)

- Minimum (less than €12,500)
- Below average (€12,500 – €26,000)
- Average (€26,000 – €39,000)
- 1 to 2 times average (€39,000 – €65,000)
- 2 times average or more (€65,000 or more)
- Don't know / Prefer not to say

On a scale from 0 to 10, how often do you generally have social contact with family, friends, neighbors, or acquaintances?

| 0 (no contact at all) | 1 | 2 | 3 | 4 | 5 | 6 | 7 | 8 | 9 | 10 (very frequent contact) |
| --- | --- | --- | --- | --- | --- | --- | --- | --- | --- | --- |
| o | o | o | o | o | o | o | o | o | o | o |

***2. Heat-related discomfort***

On a scale from 0 to 10, to what extent did you experience discomfort due to the recent heat?

| 0 (no discomfort at all) | 1 | 2 | 3 | 4 | 5 | 6 | 7 | 8 | 9 | 10 (extreme discomfort) |
| --- | --- | --- | --- | --- | --- | --- | --- | --- | --- | --- |
| o | o | o | o | o | o | o | o | o | o | o |

This question is about how you felt mentally during the recent heat. For each statement, indicate how you felt compared to normal.

|  | Much less | Less | Similar | More | Much more | I don’t know |
| --- | --- | --- | --- | --- | --- | --- |
| Gloomy/downhearted |  |  |  |  |  |  |
| Happy |  |  |  |  |  |  |
| Calm and relaxed |  |  |  |  |  |  |
| Nervous |  |  |  |  |  |  |
| Irritated |  |  |  |  |  |  |

***3. Heat-related health issues***

On a scale from 0 to 10, how concerned were you about your health during the recent heatwave?

| 0 (not concerned at all) | 1 | 2 | 3 | 4 | 5 | 6 | 7 | 8 | 9 | 10 (extremely concerned) |
| --- | --- | --- | --- | --- | --- | --- | --- | --- | --- | --- |
| o | o | o | o | o | o | o | o | o | o | o |

*Only for participants who previously reported chronic illness(es).* On a scale from 0 to 10, to what extent did the heat increase health issues related to your chronic condition(s)?

| 0 (no increase at all) | 1 | 2 | 3 | 4 | 5 | 6 | 7 | 8 | 9 | 10 (very strong increase) |
| --- | --- | --- | --- | --- | --- | --- | --- | --- | --- | --- |
| o | o | o | o | o | o | o | o | o | o | o |

To what extent did you experience the following health issues during the recent heatwave, compared to normal?

|  | Much less | Less | Similar | More | Much more | Not applicable |
| --- | --- | --- | --- | --- | --- | --- |
| Fatigue | o | o | o | o | o | o |
| Sleep problems | o | o | o | o | o | o |
| Concentration problems | o | o | o | o | o | o |
| Dizziness | o | o | o | o | o | o |
| Headaches | o | o | o | o | o | o |
| Skin irritation / itchiness | o | o | o | o | o | o |
| Muscle cramps | o | o | o | o | o | o |
| Nausea | o | o | o | o | o | o |
| Fainting | o | o | o | o | o | o |
| Heart problems | o | o | o | o | o | o |
| Respiratory problems | o | o | o | o | o | o |
| Swollen ankles/legs/feet/hands | o | o | o | o | o | o |
| Dehydration | o | o | o | o | o | o |
| Heatstroke | o | o | o | o | o | o |
| Other, namely: [Open field] | o | o | o | o | o | o |

Did you seek medical help due to (worsened) health issues during the heat?

- Yes, due to [Open field]
- No
- Prefer not to say

***4. Protective measures taken***

On a scale from 0 to 10, to what extent did you take measures to prevent or reduce health complaints during the recent heat?

| 0 (no measures at all) | 1 | 2 | 3 | 4 | 5 | 6 | 7 | 8 | 9 | 10 (a lot of measures) |
| --- | --- | --- | --- | --- | --- | --- | --- | --- | --- | --- |
| o | o | o | o | o | o | o | o | o | o | o |

How often did you take the following actions during the recent heatwave *to cool the home*?

|  | Never | Rarely | Sometimes | Regularly | Often |
| --- | --- | --- | --- | --- | --- |
| Using a fan | o | o | o | o | o |
| Using a portable air conditioner | o | o | o | o | o |
| Using a fixed air conditioner | o | o | o | o | o |
| Using a heat pump for cooling | o | o | o | o | o |
| Closing sunshades or roller shutters | o | o | o | o | o |
| Closing curtains | o | o | o | o | o |
| Closing windows/doors during the hottest part of the day | o | o | o | o | o |
| Opening windows/doors when it’s cooler outside | o | o | o | o | o |
| Other, namely: [Open field] | o | o | o | o | o |

How often did you take the following actions during the recent heatwave *to cool yourself*?

|  | Never | Rarely | Sometimes | Regularly | Often |
| --- | --- | --- | --- | --- | --- |
| Spraying water on your skin | o | o | o | o | o |
| Soaking hands or feet in cold water | o | o | o | o | o |
| Taking a cold shower or bath | o | o | o | o | o |
| Using a cooling vest or cooling pack | o | o | o | o | o |
| Spraying water on your skin | o | o | o | o | o |
| Other, namely: [Open field] | o | o | o | o | o |

How often did you take the following actions during the recent heatwave *to prevent dehydration*?

|  | Never | Rarely | Sometimes | Regularly | Often |
| --- | --- | --- | --- | --- | --- |
| Drinking more fluids (non-alcoholic) | o | o | o | o | o |
| Drinking cold water | o | o | o | o | o |
| Eating more ice cream/popsicles | o | o | o | o | o |
| Drinking sports drinks or ORS solutions | o | o | o | o | o |
| Other, namely: [Open field] | o | o | o | o | o |

How often did you take the following actions during the recent heatwave *(other)*?

|  | Never | Rarely | Sometimes | Regularly | Often |
| --- | --- | --- | --- | --- | --- |
| Changing clothing choices (e.g., lighter, thinner, breathable clothes) | o | o | o | o | o |
| Staying indoors during the hottest part of the day | o | o | o | o | o |
| Seeking shade when outdoors | o | o | o | o | o |
| Adjusting daily activities to reduce physical exertion | o | o | o | o | o |
| Seeking out cool indoor spaces (e.g., library, friend’s home with AC) | o | o | o | o | o |
| Seeking cool outdoor places (e.g., shaded park) | o | o | o | o | o |
| Cooling off in a pool or natural water (lake, river, sea) | o | o | o | o | o |
| Reducing alcohol consumption (if applicable) | o | o | o | o | o |
| Other, namely: [Open field] | o | o | o | o | o |

***5. Perceptions***

To what extent do you agree with the following statement?

|  | Strongly disagree | Disagree | Neither agree nor disagree | Agree | Strongly agree | Not applicable |
| --- | --- | --- | --- | --- | --- | --- |
| I am at greater risk of health problems from heat than other people. | o | o | o | o | o | o |
| There is little that can actually be done to prevent or reduce health complaints during a heatwave. | o | o | o | o | o | o |
