# Supplementary material for: Comparing heatwave experiences, behaviors, and risk perceptions across high-risk populations in the Netherlands: A cross-sectional survey study
Source: J Clim Chang Health. 2026 May 16;29:100689. doi: 10.1016/j.joclim.2026.100689 (PMC13199810; doi:10.1016/j.joclim.2026.100689)
Supplement: Supplementary file 2 [file mmc2.docx]

**Supplementary Material 2 – Coding of Variables and Data Analysis**

***1. Coding of risk groups***

| **Variable** | **Survey question(s) and coding** | **High risk** | **Low risk** | **NA** |
| --- | --- | --- | --- | --- |
| Age | *“What is your age (in years)?”* | ≥65 | <65 |  |
| Gender | *“What is your gender?”*  1 = Male  2 = Female | 2 | 1 |  |
| Health Status | Q1. *“How would you describe your general health?”* (1 = Excellent to 5 = Poor; 6 = Prefer not to say) Q2. *“Do you have one or more chronic illnesses?”* (coded per condition as 0 = No, 1 = Yes) Q3. *“Which of the following medications do you use?”* (coded per medication as 0 = No, 1 = Yes) | • Q1 = “Fair” or “Poor” (4 or 5), AND/OR • ≥1 chronic illness, AND/OR • ≥1 relevant medication | All others |  |
| Socio-economic position (SEP) | *“What is your household’s gross annual income?”* 1 = < €12,500  2 = €12,500–€26,000  3 = €26,000–€39,000  4 = €39,000–€65,000  5 = > €65,000  6 = Don’t know / Prefer not to say | 1-2 | 3-5 | 6 |
| Social isolation | *“On a scale from 0 to 10, how often do you generally have social contact with family, friends, neighbors, or acquaintances?”* | ≤3 | >3 |  |

***2. Coding of outcomes***

| **Outcome** | **Survey Question** | **Scale / Response Options** | **Coding** |
| --- | --- | --- | --- |
| Overall heat-related discomfort | “On a scale from 0 to 10, to what extent did you experience discomfort due to the recent heat?” | 0 (no discomfort at all) to 10 (extreme discomfort) | Treated as continuous variable |
| Emotional response | “This question is about how you felt mentally during the recent heat. For each statement, indicate how you felt compared to normal.”  Emotions: Gloomy/downhearted, Happy, Calm/relaxed, Nervous, Irritated | Much less, Less, Similar, More, Much more, I don’t know | Recoded to binary:  1 = More or Much more  0 = all others |
| **Outcome** | **Survey Question** | **Scale / Response Options** | **Coding** |
| Concern about heat-related health issues | “On a scale from 0 to 10, how concerned were you about your health during the recent heatwave?” | 0 (not concerned at all) to 10 (extremely concerned) | Treated as continuous variable |
| Worsening chronic condition | “To what extent did the heat increase health issues related to your chronic condition(s)?” | 0 (no increase at all) to 10 (very strong increase) *(Only for respondents with chronic conditions)* | Treated as continuous variable |
| Specific heat-related health issues | “To what extent did you experience the following health issues during the recent heatwave, compared to normal?”  Issues: Fatigue, Sleep problems, etc. See Supplementary Materials 1 for full list. | Much less, Less, Similar, More, Much more, Not applicable | Recoded to binary:  1 = More or Much more  0 = all others |
| Seeking medical help | “Did you seek medical help due to (worsened) health issues during the heat?” | Yes, No | Treated as binary variable |
| Extent of protective measures taken | “On a scale from 0 to 10, to what extent did you take measures to prevent or reduce health complaints during the recent heat?” | 0 (no measures at all) to 10 (a lot of measures) | Treated as continuous variable |
| Specific protective measures | “How often did you take the following actions during the recent heatwave?”  Measures: Using fan, AC, drinking fluids, etc. See Supplementary Material 1 for full list. | Never, Rarely, Sometimes, Regularly, Often | Treated as ordinal variable |
| Perceived heat risk | “I am at greater risk of health problems from heat than other people.” | Strongly disagree, Disagree, Neither, Agree, Strongly agree, Not applicable | Recoded to binary:  1 = Agree or Strongly agree  0 = all others |
| Perceived effectiveness of protective measures | “There is little that can actually be done to prevent or reduce health complaints during a heatwave.” | Strongly disagree, Disagree, Neither, Agree, Strongly agree, Not applicable | Recoded to binary:  1 = Agree or Strongly agree  0 = all others |

***3. Unadjusted differences between low- and high-risk groups for heat-related outcomes***

- For **continuous outcomes**, independent samples t-tests were used to compare means between low- and high-risk groups.
- For **binary outcomes**, Pearson’s **Chi-square tests** were conducted.
- For **ordinal outcomes**, **Mann–Whitney U tests** were used.

***4. Adjusted differences between low- and high-risk groups for heat-related outcomes***

- To assess the independent contribution of each risk factor, **multivariable regression models** were estimated including all five risk indicators as predictors.
- The type of regression analysis was based on the scale of the dependent variable:
  - **Multivariate linear regression** for continuous outcomes
  - **Multivariate binary logistic regression** for binary outcomes
  - **Multivariate ordinal logistic regression** for ordinal outcomes

***5. Association between multiple-risk index and heat-related outcomes***

- A **multiple-risk index** was computed by summing the number of high-risk characteristics, resulting in a score ranging from 0 to 5.
- **Univariate regression models** were used to examine associations between this index and each heat-related outcome.
  - **Univariate linear regression** for continuous outcomes
  - **Univariate binary logistic regression** for binary outcomes
  - **Univariate ordinal logistic regression** for ordinal outcomes

***6. Association between perception variables and heat-related outcomes***

- Two **perception variables** were examined:

1. “I am at greater risk of health problems from heat than others.”
2. “There is little that can be done to prevent or reduce heat-related health issues.”

- Both were treated as **binary independent variables**, and separate **multivariable logistic regression models** were estimated for each outcome. All models were adjusted for the five individual risk factors.

*In cases where assumptions for parametric tests were violated, appropriate robust standard errors or non-parametric alternatives were applied.*
