# Supplementary material for: Comparing heatwave experiences, behaviors, and risk perceptions across high-risk populations in the Netherlands: A cross-sectional survey study
Source: J Clim Chang Health. 2026 May 16;29:100689. doi: 10.1016/j.joclim.2026.100689 (PMC13199810; doi:10.1016/j.joclim.2026.100689)
Supplement: Supplementary file 3 [file mmc3.docx]

**Supplementary Material 3 – Representativeness of the Study Sample**

The target group of this study consisted of residents of Limburg aged 50 years and older. To define the study population, a sample was drawn from a ISO-certified Flycatcher panel. This sample was stratified by gender, age, education level, and region. This means that the individuals in the sample formed a representative reflection of Limburg residents aged 50 and older based on these characteristics, as shown in the tables below. Background information is known for all panel members and has been updated at least once in the year prior to the survey.

The statistics used to determine representativeness are based on the so-called "Golden Standard," a benchmarking tool specifically developed by the Data & Insights Network (formerly MOA) in collaboration with Statistics Netherlands (CBS) (<https://datainsightsnetwork.nl/advies-en-tools/gouden-standaard/>).

Note: due to rounding, the sum of the numbers may not always equal the total.

| **Gender** | **Response** | **CBS 2022** |
| --- | --- | --- |
| Male | 48% | 49% |
| Female | 52% | 51% |
| Total | 100% | 100% |

| **Age** | **Response** | **CBS 2022** |
| --- | --- | --- |
| 50-54 years | 15% | 16% |
| 55-59 years | 16% | 18% |
| 60-64 years | 16% | 16% |
| 65 years and older | 53% | 51% |
| Total | 100% | 100% |

| **Education level** | **Response** | **CBS 2022** |
| --- | --- | --- |
| Low | 41% | 39% |
| Middle | 38% | 40% |
| High | 20% | 22% |
| Total | 100% | 100% |

| **Geographical region** | **Response** | **CBS 2022** |
| --- | --- | --- |
| North | 24% | 23% |
| Central | 21% | 18% |
| South | 55% | 58% |
| Total | 100% | 100% |
